# Chitotriosidase and Neopterin as Potential Biomarkers for the Evaluation of Complicated Cholecystitis—A Pilot Study

**DOI:** 10.3390/jcm12041641

**Published:** 2023-02-18

**Authors:** Vlad-Ionuţ Nechita, Nadim Al Hajjar, Cristina Drugan, Cristina-Sorina Cătană, Emil Moiş, Mihaela-Ancuţa Nechita, Florin Graur

**Affiliations:** 1 Department of Medical Informatics and Biostatistics, Iuliu Hațieganu University of Medicine and Pharmacy, Louis Pasteur Str. No. 6, 400349 Cluj-Napoca, Romania; 2 Octavian Fodor Regional Institute of Gastroenterology and Hepatology, 400162 Cluj-Napoca, Romania; 3 Department of Surgery, Iuliu Hațieganu University of Medicine and Pharmacy, Croitorilor Str. No. 19–21, 400162 Cluj-Napoca, Romania; 4 Department of Medical Biochemistry, Iuliu Haţieganu University of Medicine and Pharmacy, Louis Pasteur Str. No. 6, 400349 Cluj-Napoca, Romania; 5 Ion Chiricuță Oncology Institute, Republicii Str. No. 34–36, 400015 Cluj-Napoca, Romania

**Keywords:** complicated cholecystitis, laparoscopic cholecystectomy, neopterin, chitotriosidase

## Abstract

Gallstones are a common surgical pathology. Laparoscopic cholecystectomy represents the elective treatment. Complicated cases can increase the rate of conversion, the duration, and the difficulty of the intervention, along with the hospitalization period. A prospective cohort study was conducted on 51 patients with gallstones. Only subjects with normal renal, pancreatic, and hepatic functions were included. The severity of cholecystitis was evaluated by considering the ultrasound examination, intraoperative findings, and pathology report. We evaluated two potential biomarkers, namely neopterin and chitotriosidase, by comparing their levels before and after the intervention for chronic (*n* = 36) and complicated (*n* = 15) cases, as well as their eventual association with the hospitalization period. Subjects with complicated cholecystitis had significantly higher (*p* = 0.01) neopterin levels at presentation (16.82 nmol/L vs. 11.92 nmol/L, median values), but the differences in chitotriosidase activity between complicated (170.00 nmol/mL/h) and chronic (160.00 nmol/mL/h) cases were not significant (*p* = 0.66). Patients with neopterin levels above the cut-off value 14.69 nmol/L had a 3.34 times higher risk of complicated cholecystitis. Twenty-four hours after the laparoscopic cholecystectomy, the differences in neopterin level and chitotriosidase activity between chronic and complicated cases were not significant. A significant decrease in chitotriosidase activity was observed after the intervention, only for complicated cases (190 nmol/mL/h vs. 145 nmol/mL/h, *p* = 0.007); for neopterin, the postoperative decrease was not statistically significant (19.42 nmol/L vs. 10.92 nmol/L, *p* = 0.06). No significant association with the hospitalization period was observed. Neopterin may be a useful biomarker for complicated cholecystitis, and chitotriosidase may have prognostic utility in early patient follow-up.

## 1. Introduction

Cholecystitis represents a common surgical pathology; about 10–15% of the adult population in industrialized countries will develop gallstones during their life [1,2,3], but about 50–70% will remain asymptomatic until diagnosis [3]. This pathology is twice as frequent in females, and the prevalence increases for both sexes with older age [1,4]. The treatment of choice for gallstones is laparoscopic cholecystectomy, which is less invasive than the classical procedure, while the hospitalization and recovery periods are shorter [5]. Between 1 and 2% of asymptomatic gallstone cases develop complicated cholecystitis each year [6]. About 2–30% lead to gangrenous cholecystitis, an advanced stage of inflammation with a risk of perforation and increased mortality [7,8]. A positive Murphy sign, gallbladder inflammation, wall thickening, and pericholecystic fluid can be visualized using ultrasonography in acute cholecystitis, with irregular wall thickness and intramural echoes without shadow, respectively, and intramural gas for gangrenous cases [8].

Chitotriosidase and neopterin are being increasingly considered as markers of cellular immune system activation. They are secreted by activated macrophages, which play an essential role in inflammatory response [9]. Chitotriosidase is an enzyme belonging to the family of chitinases [9,10], and, as such, it plays an important role in human protection against chitin cell-wall pathogens [10]. Nowadays, it is being considered as an enzyme involved in the innate immune response [10]. Its expression and production are stimulated by GM-CSF (granulocyte-macrophage colony-stimulating factor), lipopolysaccharides, TNF-α (tumor necrosis factor-alpha), and IFN-γ (gamma-interferon) [11,12]. Several studies have documented the usefulness of circulating chitotriosidase as a biomarker of macrophage activation in many inflammatory, autoimmune, or genetic diseases, reflecting disease severity and evolution [13]. Chitotriosidase, encoded by the *CHIT1* gene, was the first chitinolytic enzyme discovered in humans. The gene is located on chromosome 1q31-q32 and has two isoforms, which generate the molecules of 50 and 39 kDa [14]. The expression of *CHIT1* takes place mostly in activated macrophages, but also in neutrophils or Kupffer cells [15].

Neopterin belongs to the class of pteridines and is a product of GTP (guanosine triphosphate) catabolism [9,16]. Pteridines are heterocyclic chemical compounds that result from the fusion between pyrazine and pyrimidine rings [17]. The molecular structure of neopterin is [*D*-erythro-6-(1′,2′,3′-trihydroxypropyl)-pterin], with a molecular mass of 253 Da [16]. Monocyte and macrophage activation through gamma-interferon can lead to the release of neopterin [16,18,19] after T cell stimulation with IL-2 (interleukin-2), GM-CSF, TNF-α, and lipopolysaccharides. IFN-γ upregulates the enzyme cyclohydrolase-1 in macrophages, resulting in a conversion of GTP to 7.8-dihydroneopterin-triphosphate, which, after hydrolysis by a phosphatase, is transformed to 7.8-dihydroneopterin. Furthermore, neopterin is generated after oxidation by superoxide hypochlorite [20]. Neopterin and its derivatives can increase the cytotoxic activity of dendritic cells and activated macrophages and its synthesis is associated with oxidative stress [16,20]. Elevated neopterin concentrations in blood or urine can reflect endogenous release of gamma-interferon and cellular immunity activation, namely the activation of T-cells and macrophages [16,21]. Murr et al. suggested that neopterin concentrations may be used to detect non-specific acute infections in blood donors, enabling safe transfusion. They may also be used to track the efficiency of therapy in autoimmune diseases or HIV (human immunodeficiency virus) infections [18].

In surgery, these molecular biomarkers were evaluated for acute appendicitis, and they were relevant to diagnosis and disease severity. Acar et al. observed higher chitotriosidase activity in patients with acute appendicitis [22]. Coşkun et. al. evaluated neopterin as a biomarker of acute appendicitis in rabbits, with a cut-off level at 34.475 nmol/L [23]. Kamal et al. suggested that neopterin might be a useful biomarker in acute appendicitis for humans, with better sensitivity than the Alvarado score ≥ 7, at a cut-off level of 5.3 nmol/L [24]. Furthermore, Dal et al. evaluated neopterin for 100 patients with suspected acute appendicitis, finding significantly higher values in complicated cases [25].

Our study focused on the evaluation of these biomolecules as potential biological markers in patients with cholecystitis based on the inflammatory response associated with this condition. Therefore, we aimed to evaluate the differences in chitotriosidase activity and neopterin levels between simple chronic and complicated cholecystitis at presentation and during early postoperative recovery. We also investigated the relationship between the levels of these biomarkers at patient presentation and the total hospitalization period, the postoperative hospitalization period, and the eventual requirement of additional maneuvers during the procedure, i.e., the invasiveness of the surgical approach.

## 2. Materials and Methods

### 2.1. Setting and Study Design

We conducted an observational prospective, longitudinal cohort study on patients with cholecystitis or those presenting with gallstones at “Prof. Dr. Octavian Fodor” Regional Institute of Gastroenterology and Hepatology, Cluj-Napoca, Romania, between August 2019 and January 2021.

The study received approval from the “Iuliu Hațieganu” University Ethics Committee (No. 121/24 April 2019) and approval from the Ethics Committee of the “Prof. Dr. Octavian Fodor” Regional Institute of Gastroenterology and Hepatology (No. 8900/10 July 2019) before patients’ enrollment.

### 2.2. Participants

Patients with an ultrasound-confirmed diagnosis of gallstones that had an indication for laparoscopic cholecystectomy and gave their informed consent for participation were included in the study.

The sample was divided into two major subgroups, namely chronic and complicated cholecystitis, according to the ultrasound examination (gallstones, positive Murphy sign, wall thickening > 4 mm, sonolucent layer in the gallbladder wall, intramural gas, enlarged gallbladder, and presence of pericholecystic fluid) [8,26], the intraoperative findings (chronic, catarrhal, phlegmonous, gangrenous, pyocholecyst, or perforated forms), and the histopathology report (inflammation, ulcerations, and erosions). The most important factor in deciding the type of cholecystitis was intraoperative diagnosis. To state the type of cholecystitis, a positive intraoperative diagnosis was sufficient, even without a relevant ultrasonography or pathology report.

The following exclusion criteria were considered: abnormal renal function, biliary passage, cholestasis, cholangitis, biliary pancreatitis, elevated liver enzymes, conversion to the classical surgical procedure, and blood loss of more than 100 mL during the intervention. The biochemistry parameters for blood coagulation and electrolytes were in the normal range for all included subjects.

### 2.3. Data Source and Collection

For each patient, demographic data (age, and sex), routine blood test results (complete blood count, liver, kidney, and pancreatic function tests), preoperative ultrasound examination, intraoperative findings, and information about the surgical procedure, as well as histopathology results, were collected.

For neopterin and chitotriosidase determinations, blood samples were collected in 4 mL EDTA vacutainers for each patient at presentation and then in the first 24 h after surgery. The samples were centrifuged within 10 to 15 min after collection at 3000 rpm and 4 °C for 10 min. The plasma separated in the upper phase was stored for further processing at −20 °C. Neopterin quantification was performed using the Human Neopterin ELISA kit (Wuhan Fine Biotech, Wuhan, China) according to the instructions of the manufacturer. Plasma chitotriosidase activity was measured using an artificial fluorescent substrate (4-methyl-umbelliferyl-chitotrioside), according to the previously described method [27], and the results were expressed as nanomoles of hydrolyzed substrate per milliliter per hour (nmol/mL/h).

### 2.4. Statistical Methods

For the statistical analysis, R version 4.0.5 with R Commander was used (R Foundation for Statistical Computing, Vienna, Austria). To evaluate quantitative data distribution, we used the Shapiro–Wilk test, skewness, and kurtosis. Quantitative data were presented as mean ± standard deviation (in case of normal distribution) or median and interquartile range (for non-normal distribution). We used the Chi-square test to compare frequencies and Fisher’s Exact test when the expected frequencies were under 5. The relative risk was used to compare the risks between the groups. Mann–Whitney tests for independent groups and the Wilcoxon test for paired groups were used when we compared quantitative data. A log-rank test compared differences for the hospitalization period. To find the optimal cut-off values and the classification ability of the biomarkers to predict cholecystitis severity and drainage, we used the Receiver Operating Characteristics (ROC) curve, along with the maximum Youden index. The area under the curve (AUC) with 95% confidence intervals computed with bootstrap was presented. A *p*-value less than 0.05 was considered statistically significant.

We hypothesized that preoperative neopterin level and chitotriosidase activity were different between chronic and complicated cases, that they were related to the necessity for additional invasive maneuvers during the surgical procedure (adhesiolysis, drainage placement, and aponeurosis enlargement), and that their values would decrease early (during the first 24 h) after the intervention.

## 3. Results

Fifty-three patients that signed informed consent were considered for evaluation; two of them were excluded (one refused the intervention afterward, and the other proved to have elevated hepatic and pancreatic biochemistry samples). Fifty-one patients with gallstones were included in the study. According to the severity of cholecystitis, we had two groups: thirty-six subjects with chronic uncomplicated cholecystitis (36/51, 70.58%) and fifteen complicated cases (15/51, 29.41%). In the group with complicated cases, one patient presented with a pyocholecyst (6.6%), three with catarrhal cholecystitis (20%), four with gangrenous cholecystitis (26.66%), and seven with phlegmonous forms (46.66%). In the sample, there were 37 females (72.54%) with a female-to-male ratio of 2.64 (37/14) and the average age for the sample was 51.13 ± 15.85 years old. For the group with complicated cases, the average age was 54.8 ± 18.06, with 9 (60%) females; in the group with chronic cases, the average age was 49.61 ± 14.85, with 28 (78%) females. The white blood cells varied between 3.68 and 17.23 × 10^3^/mm^3^ and neutrophils level between 2.11–12.36 × 10^3^/mm^3^ in the studied cohort.

A significantly higher number of white blood cells, neutrophils, and a significantly higher neopterin level were observed at the presentation for subjects with complicated cholecystitis, but the differences in chitotriosidase activity were not statistically significant between complicated and uncomplicated, chronic cases. The liver and pancreatic enzymes were comparable (Table 1) between the simple and complicated groups. The differences were also significant regarding the surgery duration and hospitalization period (Table 1), but on the first day after laparoscopic intervention, both neopterin level and chitotriosidase activity were not statistically significantly different between the groups (Table 1).

Neopterin levels and chitotriosidase activity at a patient’s presentation were not significantly different for those that required a more invasive surgical procedure (adhesiolysis, aponeurosis enlargement, or postoperative drainage) in comparison with the groups who did not undergo these procedures. No significant differences were detected for baseline white blood cells and neutrophils. Similarly, the first postoperative day after laparoscopic cholecystectomy, no differences were observed for neopterin level nor for chitotriosidase activity between the groups with or without adhesiolysis, drainage, or aponeurosis enlargement (Table 2). Additionally, no significant differences in the white blood cell count or the neutrophil count were observed according to the adjacent procedures.

When comparing the preoperative with the early postoperative values, no significant changes were observed for neopterin [14.89 (10.48–21) nmol/L vs. 12.25 (8.84–20.62) nmol/L] after the laparoscopic intervention, but there was a significant reduction (*p* = 0.003—Wilcoxon test) in the chitotriosidase activity 160 (105–225) nmol/mL/h vs. 140 (110–195) nmol/mL/h]. No significant differences were observed in the first postoperative day for neopterin regarding some additional invasive maneuvers: sub-hepatic drainage, aponeurosis enlargement, or adhesiolysis. A significant decrease in chitotriosidase activity was observed after the intervention for complicated cases, with drainage and aponeurosis enlargement (Table 3).

**Figure 1:**
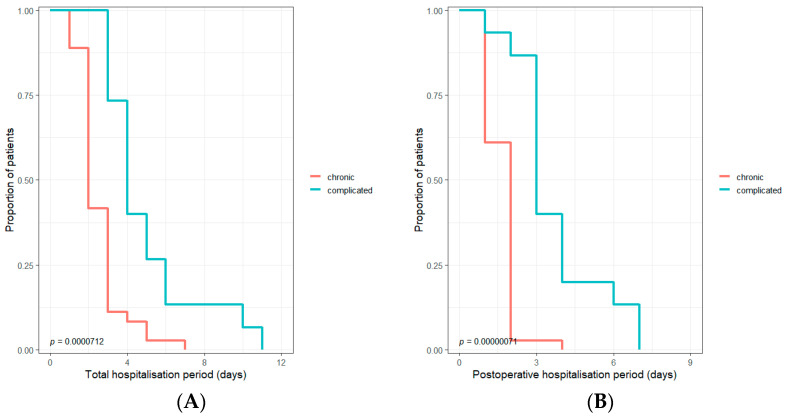
The Kaplan–Mayer curve to compare total (**A**) and postoperative (**B**) hospitalization period for chronic vs. complicated cholecystitis.

No statistically significant differences were observed for the group that had neopterin levels above the median value of 13.03 nmol/L in comparison with lower neopterin levels regarding the total hospitalization period (*p* = 0.35—Mann–Whitney), nor for days of hospitalization after the surgery (*p* = 0.13—Mann–Whitney). The significance was also absent when we compared the group with chitotriosidase activity above the median value of 170 nmol/mL/h with those below this value, regarding the total hospitalization period (*p* = 0.65—Mann–Whitney) and also the post-operative hospitalization period (*p* = 0.72—Mann–Whitney). A significant difference in the hospitalization period was observed between chronic and complicated cholecystitis (Figure 1).

**Figure 2:**
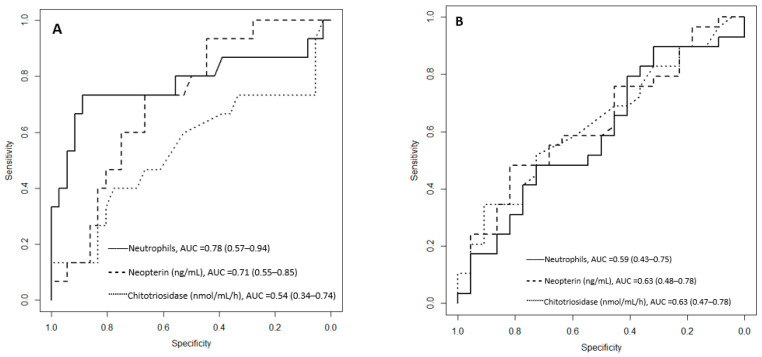
ROC curves (Receiver Operating Characteristic) for neopterin level and chitotriosidase activity considering complicated cases (**A**) and the necessity for subhepatic drainage (**B**). AUC = area under the curve, with 95% confidence intervals.

After performing a ROC (Receiver Operating Characteristic) curve for complicated cholecystitis, the following cut-off values were observed: 6.2 × 10^3^ for neutrophils number; 14.69 nmol/L for neopterin; and 225 nmol/mL/h for chitotriosidase activity. For the necessity of subhepatic drainage, the cut-off values were: 3.18 for neutrophils number, 16.38 for neopterin, and 235 nmol/mL/h for chitotriosidase activity. The corresponding AUC (area under the curve) and 95% confidence intervals are presented in Figure 2.

For the patients with neopterin levels above the cut-off value (>14.69 nmol/L), the risk of complicated cholecystitis was 3.34 times higher (95%CI 1.16–11.42; *p* = 0.013—Fisher Exact test). For the patients with chitotriosidase activity above the cut-off value (>225 nmol/mL/h), the risk of complicated cholecystitis was 1.76 times higher (95%CI 0.62–4.2; *p* = 0.16—Chi-square test).

For the patients with neopterin levels above the cut-off value (>16.38 nmol/L), the subhepatic drainage was used 1.71 times more frequently (95%CI 1.13–2.57; *p* = 0.047—Fisher Exact test). For the patients with chitotriosidase activity above the cut-off value (>235 nmol/mL/h), the subhepatic drainage was used 1.72 times more frequently (95%CI 1.09–2.67; *p* = 0.0.038—Fisher’s Exact test).

## 4. Discussion

Our study evaluated two novel, infrequently used inflammatory biomarkers in cholecystitis, a common surgical pathology. Complicated cholecystitis may increase the difficulty and duration of laparoscopic cholecystectomy, inducing a longer postoperative hospitalization period.

The sample was representative of the studied pathology: the number of females was about two times higher than that of males [1,4]. To eliminate the biases, subjects with abnormal kidney function and increased liver or pancreatic enzymes were excluded. For these analytes, the median values for the studied sample and its subgroups are presented in Table 1, indicating no statistically significant differences between simple and complicated cholecystitis. White blood cells and neutrophil counts are probably the laboratory findings most frequently used to evaluate a patient’s inflammatory status, to differentiate between chronic and complicated cholecystitis [26,28], and to decide the necessity of emergency surgical intervention. Likewise, among our patients, significantly higher values were observed for complicated cases (Table 1).

We did not find previously published studies addressing the usefulness of neopterin or chitotriosidase in biliary pathology. Coşkun et al. evaluated neopterin in acute appendicitis on a rabbit model [23]. They suggested an optimal cut-off at 34.475 nmol/L. Rabbits with neopterin levels above 34.475 nmol/L had a higher probability (4.66 times) of acute appendicitis. Dal et al. evaluated neopterin along with other common inflammatory biomarkers in 100 patients presented in the emergency department with suspicion of acute appendicitis [25]. No significant differences (*p* = 0.107—Mann–Whitney test) were observed between patients with (*n* = 60) and those without (*n* = 40) acute appendicitis, but significantly higher neopterin levels were observed in 11 complicated appendicitis cases in comparison with 49 uncomplicated cases (*p* = 0.049—Mann–Whitney test). For the complicated cases, a significantly higher preoperative neopterin level was also observed in our study, but for the preoperative chitotriosidase activity, the differences did not reach statistical significance (Table 1). On the other hand, Acar et al. evaluated chitotriosidase activity in thirty-four patients with appendectomies [22]. Significantly higher (*p* < 0.05—Mann–Whitney test) chitotriosidase activity was found in the group with acute appendicitis (99.7 ± 14.4 nmol/mL/h), compared to those that had normal appendixes (86.2 ± 12.3 nmol/mL/h), according to the pathology report.

At twenty-four hours after the laparoscopic gallbladder removal, the differences in biomarker levels between chronic uncomplicated and complicated cholecystitis were statistically insignificant (Table 1). Concerning the values before the intervention, no statistically significant differences (*p* > 0.05—Mann–Whitney test) were noted between patients without and those who needed additional procedures during an intervention, such as adhesiolysis, drainage, or aponeurosis enlargement (Table 2). This pattern of insignificant variation was also observed for the white blood cell and neutrophil counts, as well as the new inflammatory biomarkers. We must mention that aponeurosis enlargement is more related to gallstone dimensions than to the inflammatory process itself, but adherences can be related to surgical history, previous inflammatory responses caused by repeated biliary episodes, and to complicated cholecystitis [29]. The drainage maneuver can also be influenced by surgeons’ preferences; surgeons may use subhepatic drainage for some uncomplicated cases.

The presence of complicated cases explains the differences in surgery duration and hospitalization period (Table 1). When comparing the total hospitalization period and the post-operative hospitalization period between patients with neopterin levels or chitotriosidase activity above the median value of the studied sample to those with lower values of these biomarkers, no significant differences were observed. According to our results, these differences were only significant regarding the type of cholecystitis (Figure 1).

We evaluated the neopterin and chitotriosidase activity optimal cut-off values at presentation in patients with complicated cholecystitis requiring subhepatic drainage that can increase the hospitalization period. Along with the novel biomarkers, the neutrophil cut-off value and area under the curve (AUC) were also calculated for comparison (Figure 2). In order to differentiate between chronic and complicated cases, the best AUC was observed for neutrophils, followed by neopterin levels. The AUC for chitotriosidase activity was poor, indicating its inability to differentiate accurately between uncomplicated and complicated cases.

Better results were observed for the novel biomarkers regarding the necessity of post-operative subhepatic drainage, where the AUC was lower for the neutrophil count. Kamal et al. also evaluated neopterin levels in acute appendicitis [24], presenting a cut-off value of 5.3 nmol/L, with a higher accuracy (82.1 vs. 76.2) and sensitivity (85.4 vs. 74.1) but lower specificity (76.9 vs. 80.8) than the Alvarado score ≥ 7. The AUC with 95% confidence intervals for the ROC analyses showed similar values for neopterin 0.862 (0.783–0.941) and Alvarado score 0.860 (0.783–0.937). We may assume a similar pattern could be observed for circulating neopterin levels in patients with complicated cholecystitis.

Some limits have to be considered for our research: (1) smoking habits, obesity, and other comorbidities were not considered in our evaluation, although some conditions may influence the levels of circulating inflammatory biomarkers; (2) the impact of chronic treatment was not evaluated; (3) data were collected from a single medical center; (4) the cohort was rather small and postoperative samples were available for only 35 patients.

According to our knowledge, this is the first study to evaluate neopterin and chitotriosidase levels in patients with complicated cholecystitis without renal, hepatic, and pancreatic function impairment. Our study tried to establish whether these biomarkers might have a diagnostic and prognostic value by correlating their levels with clinical evidence, other laboratory findings, and ultrasound evaluation.

## 5. Conclusions

In conclusion, circulating neopterin may be a useful molecular biomarker for the diagnosis of complicated cholecystitis, while chitotriosidase activity revealed no significant differences between patient subgroups, although it showed a significant decrease the first day after laparoscopic cholecystectomy and may have prognostic utility in the early patient follow-up. Larger cohorts are needed for the confirmation of our findings before the eventual use of these biomolecules in the clinical setting.

## Figures and Tables

**Table 1:** Sample characteristics according to the severity of cholecystitis (*n* = 51).

	All Subjects (*n* = 51)	Simple Cholecystitis (*n* = 36)	Complicated Cholecystitis (*n* = 15)	*p*-Value
White blood cells	7.34 (5.82–10.13)	7.01 (5.71–8.24)	10.91 (7.26–13.14)	0.0131
Neutrophils	4.90 (3.50–6.48)	4.29 (3.31–5.53)	7.63 (5.40–10.19)	0.00147
Chitotriosidase baseline (nmol/mL/h)	170.00 (110.00–230.00)	160.00 (110.00–212.50)	170.00 (90.00–295.00)	0.66
Neopterin baseline (nmol/L)	13.03 (7.78–18.34)	11.92 (6.79–16.39)	16.82 (13.53–21.36)	0.0177
Chitotriosidase fd * (nmol/mL/h)	140.00 (110.00–195.00)	145.00 (110.00–172.50)	140.00 (75.00–210.00)	0.82
Neopterin fd * (nmol/L)	12.26 (8.84–20.63)	13.06 (8.73–19.98)	10.94 (9.04–20.02)	0.87
AST/GOT (U/L)	22.00 (17.00–26.50)	21.50 (17.00–26.25)	24.00 (17.50–26.50)	0.766
ALT/GPT (U/L)	21.00 (16.00–28.50)	21.00 (15.75–29.25)	22.00 (16.50–27.00)	0.862
Amylase (U/L)	51.0 (42.00–63.50)	56.00 (42.00–64.25)	46.00 (39.50–57.00)	0.361
Total bilirubin (mg/dL)	0.60 (0.50–0.80)	0.60 (0.40–0.80)	0.70 (0.60–0.95)	0.119
Direct bilirubin (mg/dL)	0.27 (0.22–0.35)	0.25 (0.18–0.32)	0.31 (0.28–0.46)	0.0089
Alkaline phosphatase (U/L)	188.00 (153.00–242.00)	204.50 (161.50–242.00)	181.00 (128.50–233.50)	0.442
Gamma-glutamyl transpeptidase (U/L)	41.00 (23.00–64.50)	40.00 (22.75–61.75)	41.00 (23.00–68.00)	0.91
Surgery duration (min)	45.00 (40.00–57.50)	42.50 (35.00–55.00)	60.00 (50.00–70.00)	0.0012
Hospitalization after surgery (days)	2.00 (1.00–3.00)	2.00 (1.00–2.00)	3.00 (3.00–4.00)	0.000003
Total hospitalization (days)	3.00 (2.00–4.00)	2.00 (2.00–3.00)	4.00 (3.50–5.50)	0.000002

Values are presented as the median and interquartile range (Q1–Q3); AST/GOT = Aspartate Aminotransferase; ALT/GPT = Alanine Aminotransferase; * fd = first day after intervention, available only for 35 patients (24 chronic and 11 complicated cases); *p*-value represents the comparison between chronic and complicated cases.

**Table 2:** The association of chitotriosidase and neopterin at presentation with the invasiveness of the procedure.

Preoperative Values (*n* = 51)	Neopterin (nmol/L) Yes *	Neopterin (nmol/L) No **	*p*-Value	Chitotriosidase (nmol/mL/h) Yes *	Chitotriosidase (nmol/mL/h) No **	*p*-Value
Adhesiolisis (*n* = 23)	11.65 (6.99–20.82)	14.46 (8.45–18.09)	0.79	170 (115–400)	160 (100–205)	0.23
Drainage (*n* = 29)	15.13 (9.56–20.81)	12.52 (7.11–15.8)	0.108	180 (115–350)	160 (100–185)	0.11
Aponeurosis enlargement (*n* = 11)	9.56 (7.86–15.8)	14.69 (7.9–18.09)	0.504	160 (160–395)	170 (107.5–212.5)	0.346
Postoperative values (*n* = 35) ^#^						
Adhesiolisis (*n* = 13)	12.99 (6.52–22.63)	11.93 (9.12–17.54)	0.959	160 (110–350)	140 (110–170)	0.322
Drainage (*n* = 20)	12.64 (8.69–22.95)	11.57 (9.64–17.02)	0.8051	145 (107.5–280)	140 (115–170)	0.517
Aponeurosis enlargement (*n* = 7)	9.95 (7.78–18.17)	12.64 (9.16–19.95)	0.635	150 (130–310)	140 (107.5–187.5)	0.5093

Yes *—values for the cases that required increased invasiveness of the procedure. No **—values for the cases without adhesiolysis, drainage, or aponeurosis enlargement. ^#^ In postoperative values were available only for 35 patients (24 chronic and 11 complicated cases). Results are presented as median and quartiles.

**Table 3:** The differences at follow-up for neopterin and chitotriosidase (*n* = 35).

	Baseline Neopterin (nmol/L)	Postoperative Neopterin (nmol/L)	*p*-Value	Baseline Chitotriosidase (nmol/mL/h)	Postoperative Chitotriosidase (nmol/mL/h)	*p*-Value
All cases (*n* = 35)	14.89 (10.48–21)	12.25 (8.84–20.62)	0.22	160 (105–225)	140 (110–195)	0.00369
Chronic cholecystitis (*n* = 23)	12.94 (8.34–15.91)	12.98 (8.59–20.62)	0.78	160 (110–200)	150 (110–175)	0.07
Complicated cholecystitis (*n* = 11)	19.42 (15.07–23.35)	10.92 (9.05–20.01)	0.06	190 (90–305)	145 (75–210)	0.007
Adhesiolisis (*n* = 13)	15.25 (11.42–26.35)	12.99 (6.53–22.66)	0.27	160 (110–480)	160 (110–350)	0.11
Subhepatic drainage (*n* = 20)	16 (11.6–25.17)	12.64 (8.7–22.95)	0.29	170 (105–317.5)	145 (107.5–280)	0.04
Aponeurosis enlargement (*n* = 7)	12.17 (9.01–29.79)	9.95 (7.78–18.17)	0.17	160 (160–455)	150 (130–310)	0.04

Results are presented as median and quartiles. To compare the pre- and postoperative values, we used the Wilcoxon signed-rank test.

## Data Availability

The data presented in this study are available on request from the corresponding author. The data are not publicly available due to due to restrictions: privacy and ethical.
